# Detection of high-valent iron species in alloyed oxidic cobaltates for catalysing the oxygen evolution reaction

**DOI:** 10.1038/s41467-021-24453-6

**Published:** 2021-07-09

**Authors:** Nancy Li, Ryan G. Hadt, Dugan Hayes, Lin X. Chen, Daniel G. Nocera

**Affiliations:** 1 Department of Chemistry and Chemical Biology, Harvard University, Cambridge, MA USA; 2 Chemical Sciences and Engineering Division, Argonne National Laboratory, Lemont, IL USA; 3 Division of Chemistry and Chemical Engineering, California Institute of Technology, Pasadena, CA USA; 4 Department of Chemistry, University of Rhode Island, Kingston, RI USA; 5 Department of Chemistry, Northwestern University, Evanston, IL USA

**Keywords:** Chemistry, Energy

## Abstract

Iron alloying of oxidic cobaltate catalysts results in catalytic activity for oxygen evolution on par with Ni-Fe oxides in base but at much higher alloying compositions. Zero-field ^57^Fe Mössbauer spectroscopy and X-ray absorption spectroscopy (XAS) are able to clearly identify Fe^4+^ in mixed-metal Co-Fe oxides. The highest Fe^4+^ population is obtained in the 40–60% Fe alloying range, and XAS identifies the ion residing in an octahedral oxide ligand field. The oxygen evolution reaction (OER) activity, as reflected in Tafel analysis of CoFeO_x_ films in 1 M KOH, tracks the absolute concentration of Fe^4+^. The results reported herein suggest an important role for the formation of the Fe^4+^ redox state in activating cobaltate OER catalysts at high iron loadings.

## Abstract (web summary)

The capturing of high valent iron in a catalytic reaction is important but difficult task. Here, the authors report identification of a high-valent Fe(IV)-species with different spectroscopic tools such as Mössbauer spectroscopy and X-ray absorption spectroscopy during the course of an oxygen evolving reaction.

## Introduction

Dimensional reduction of first row metal oxides gives rise to metallate oxygen evolving catalysts (M-OECs) that exhibit high activity for the oxygen evolution reaction (OER)^1–5^. Electrodeposition of oxidic cobaltates and nickelates in the presence of phosphate and borate (CoP_i_^6,7^, CoB_i_^8,9^, NiB_i_^10,11^, MnP_i_^12,13^) results in clusters of 10–60 metal atoms, as determined from in situ pair distribution functional analysis^9,14–19^. The self-healing property of the M-OECs^2,20–22^ allows them to promote water-splitting under benign conditions. Under such conditions, the catalysts may be easily interfaced with materials for direct conversion of water to oxygen and hydrogen at high efficiency^23–26^, as well as interfaced with biological organisms to perform artificial photosynthesis^27^ at efficiencies greatly exceeding natural photosynthesis^28,29^. The metallate clusters possess a high edge-to-area ratio that engenders high activity, as revealed by isotopic labelling studies^30^ that show the critical O–O bond formation step to occur by proton-coupled electron transfer (PCET) at cluster edge sites^8,31–36^. Moreover, the electronic charge in M-OECs can delocalize within the clusters^37,38^ giving rise to electron/hole transport^39^ that can maximally couple to the ion transport needed to support the OER^40,41^.

Iron doping of metal oxide films has long been known to increase overall OER activity of metal oxide OER catalysts^42,43^. The behaviour of Fe in Ni-OECs has been revisited^44^, and the role of Fe has been ascribed to various factors, including active site Fe^4+^ or higher valent species^45–47^, near neighbour Fe effects on Ni resulting from strain on the oxide lattice^48–51^, active oxygen intermediates at Ni–Fe sites^52–54^, Fe induced partial-charge-transfer to Ni sites^55,56^, and Fe acting as a Lewis acid that promotes charge transfer character and favourable energetics for Ni oxyl formation^57,58^. Quizzically, though detected by Mössbauer spectroscopy, the presence of Fe^4+^ does not correlate with the observed catalytic activity^59^. Iron loading has also been shown to affect the OER activity of Co-OECs^60–62^, but at very different alloying loads. Whereas Ni-OECs show maximal activity with Fe loadings of ~5 mol% Fe^42,55^, the maximal activity of Co-OECs is observed for Fe loadings of >40 mol% Fe^61^. These higher loadings suggest different roles for Fe in enhancing M-OEC activity at high versus low alloying.

We now report the zero-field ^57^Fe Mössbauer and X-ray absorption spectroscopy (XAS) of Co-OEC alloyed with Fe from 0 to 100% and show that, unlike Fe alloyed in Ni-OECs, the presence of Fe^4+^ tracks OER activity, suggesting that Fe^4+^ is intimately involved as a redox activator of OER. The results suggest different roles for Fe in alloyed M-OEC catalysts. At low loadings such as in (Ni:Fe)-OECs, OER is performed by Ni active sites and Fe promotes the PCET activation of OER. At high loadings, as is observed here for Fe-alloyed Co-OEC catalysts, the redox properties of Fe appear to play a prominent and more direct role in promoting OER.

## Results

A series of CoFeO_*x*_ films with varying Fe content were prepared by cathodic deposition upon the reduction of nitrate to induce a high local basic pH near the electrode, resulting in the electrodeposition of a Co:Fe hydroxide film^13^, which was then converted to CoFeO_*x*_ with the application of an anodic potential. Metal elemental compositions were determined by inductively coupled mass spectrometry (ICP-MS) of digested films after electrochemical measurements. Previous studies have shown that Fe and Co deposit homogeneously as detected by SEM/EDS analysis^63^. As Fe content increases, both the cathodic and anodic features of the Co^2+/3+^ couple are shifted towards higher potentials (Supplementary Fig. 1). CoFeO_*x*_ films with 40–80 mol% Fe in 1 M KOH that was scrubbed of trace metal contaminants display Tafel slopes of ~30 mV/dec (Supplementary Fig. 2), similar to previously published results^61^. We note that the lowest Tafel slopes in CoFeO_*x*_ films are obtained at much higher Fe:Co ratios than observed for NiFeO_*x*_ films.

**Fig. 1:**
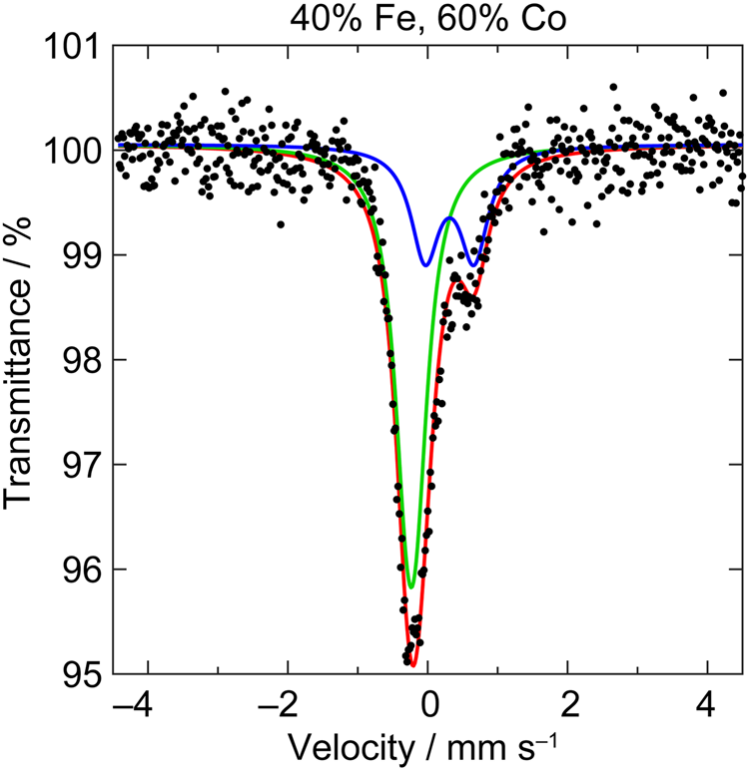
Mössbauer spectra of CoFeO_*x*_. Zero-field ^57^Fe Mössbauer spectra for CoFeO_*x*_ films with the composition 40% Fe:60% Co. Raw data (black circle), fit for Fe^3+^ species (blue line), Fe^4+^ species (green line), and overall fit (red line).

**Fig. 2:**
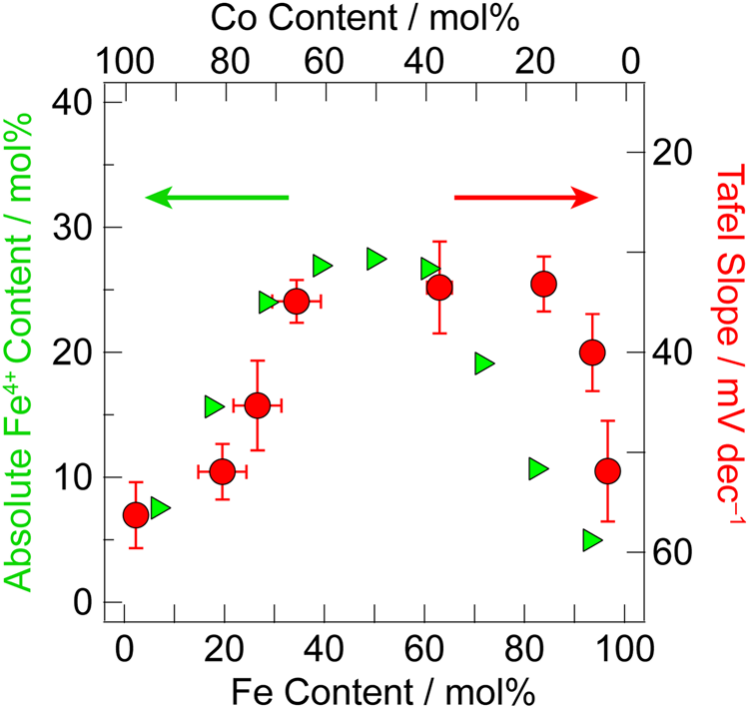
Correlation of Tafel slope with Fe^4+^ composition. Overlay of Tafel slope (red circle) with absolute Fe^4+^ (green triangle) population in CoFeO_*x*_ films with increasing Fe content. Tafel measurements were run in triplicate, and the average value is shown on the graph at 95% confidence limits.

The electronic structure of Fe centres in CoFeO_*x*_ films was probed with zero-field ^57^Fe Mössbauer spectroscopy. A representative ^57^Fe Mössbauer spectrum is given in Fig. 1; the spectra of all CoFeO_*x*_ film samples are given in Supplementary Fig. 3 and Supplementary Fig. 4. The spectra are reproducible and sensitive to Fe population changes between samples with 10 mol% Fe differences (Supplementary Fig. 5). Two species of Fe are detected in the ^57^Fe Mössbauer spectra. Fits of the spectra furnish corresponding isomer shifts (*δ*) and quadrupole splittings (|Δ*E*_Q_|) for one species with *δ* ~ 0.3 mm/s and |Δ*E*_Q_| ~ 0.7 mm/s and the other species with *δ* ~ –0.2 mm/s and |Δ*E*_Q_| less than the resolved linewidth (~0.3 mm/s). These values, which are somewhat sensitive to total Fe alloying concentrations and fittings (Supplementary Fig. 7), do not correspond to either Fe_2_O_3_^64,65^ or metallic Fe^66,67^. One species matches the Mössbauer parameters of high spin (HS) Fe^3+^ in the oxide ligand field of NiFeO_*x*_^5,68,69^ and FeOOH^70^. The Mössbauer parameters of the second Fe species correspond to those observed previously for Fe^4+^ in NiFeO_*x*_^70^, and is consistent with theoretical calculations showing the persistence of Fe^4+^ in NiFeO_*x*_^71^. The Fe^3+^:Fe^4+^ ratio, which may be determined from the Mössbauer parameters, shifts towards Fe^3+^ at low and high Fe concentrations (Supplementary Fig. 6) with a maximal absolute Fe^4+^ concentration observed between 40 and 60% Fe loading (Fig. 2). Strikingly, as Fig. 2 illustrates, the population of Fe^4+^ in CoFeO_*x*_ films tracks OER activity as reflected in Tafel slopes. We observe a direct correlation between absolute Fe^4+^ content and low Tafel slopes (30 mV/dec), implicating the important role of Fe^4+^ in enhancing OER activity in CoFeO_*x*_ films at high Fe alloying concentrations. The maximum in activity is likely a result of Fe becoming the dominant compositional metal. Unary Fe oxide films are inferior OER catalysts even as ultrathin sub-monolayer films^72^. Thus the observed maximum in activity is consistent with the active site for OER becoming dominated by an Fe-only composition at high iron loadings in excess of 50% loading.

**Fig. 3:**
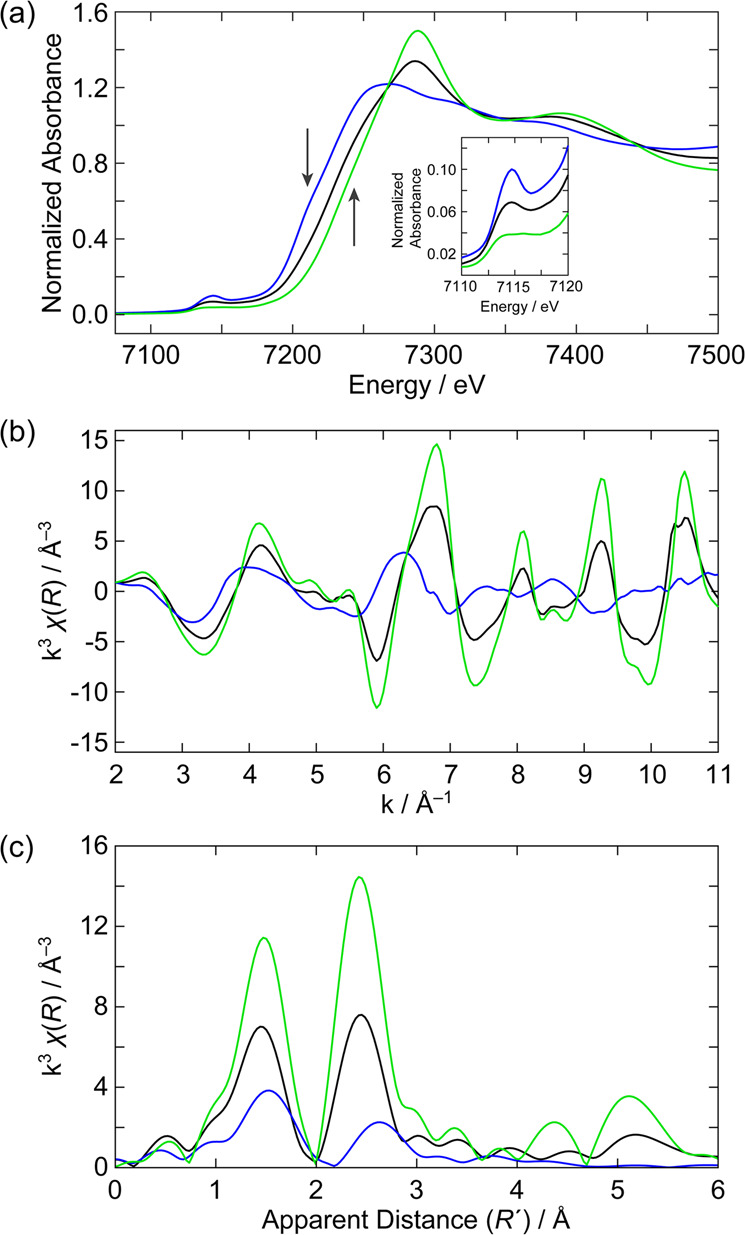
X-ray absorbance spectra of CoFeO_*x*_. **a** Fe K-edge X-ray absorbance spectra and corresponding **b** k-space and **c** R-space for CoFeO_*x*_ with the composition 50% Fe:50% Co (black line), and calculated Fe^3+^ (blue line) and Fe^4+^ spectra (green line). Inset of **a** highlights the pre-edge region.

The assignment of Fe^3+^ and Fe^4+^ as deduced by Mössbauer spectroscopy is supported by Fe K-edge XAS of CoFeO_*x*_ samples with varying Fe content. The X-ray absorption near edge structure (XANES) spectra and the k-space and R-space data are given in Fig. 3 for CoFeO_*x*_ with a 50% Fe:50% Co (black line) composition. Analogous data for several other compositions are given in Supplementary Fig. 8. As the proportions of Fe^3+^ and Fe^4+^ are known from Mössbauer, the individual XANES spectrum for each Fe^3+^/Fe^4+^ species may be ascertained from linear combination fitting. The resulting Fe^3+^ and Fe^4+^ spectra are reproducible for specific linear combinations (Supplementary Figs. 9 and  10); the spectrum for the 50% Fe:50% Co sample (black line) and corresponding linear combinations for the Fe^3+^ and Fe^4+^ (blue and green line, respectively) contributions are given in Fig. 3. Several important observations can be made between the Fe^3+^ and Fe^4+^ species. There is a large edge shift from ~7124 to ~7128 eV between the Fe^3+^ and Fe^4+^ species. Additionally, the 1*s* → 3*d* pre-edge intensity of the Fe^3+^ species is significantly higher than that for Fe^4+^ (inset of Fig. 3a). The R-space amplitude of the Fe^3+^ species is significantly lower than the Fe^4+^ species (Fig. 3c). This decreased amplitude suggests a lower coordination number. There is also a clear contraction of the first and second shell scattering distances for Fe^4+^. The pre-edge region reflects transitions to the many-electron excited states of the metal centre. The spectral intensity of the pre-edge derives from both electric quadrupole and electric dipole mechanisms. In a centrosymmetric ligand field (e.g., *O*_*h*_), the electric dipole contribution is parity forbidden, and only the quadrupole intensity is present. Conversely, deviation from centrosymmetry (e.g., *T*_*d*_) results in a significant increase in the pre-edge intensity. This increase in intensity derives from electric dipole allowedness, which tracks with the amount of 3*d*–4*p* mixing in a noncentrosymmetric ligand field^73^. These observations, together with a low coordination number from the low amplitude R-space data, suggest that Fe^3+^ is present in a distorted ligand field lacking inversion symmetry—either *T*_*d*_ or square pyramidal ligand field geometries are likely possiblities.^73^ Along similar lines for Fe^4+^, the low pre-edge intensity suggests a more symmetric *O*_*h*_ ligand field, which will largely exhibit electric quadrupole intensity. A more symmetric ligand field is also consistent with the higher amplitude R-space data and small |Δ*E*_Q_|.

The combination of Mössbauer, XAS, and EXAFS data support the assignment of a high-valent Fe^4+^ species in a symmetric *O*_*h*_ ligand field. An alternative scenario to consider is a low-spin Fe^3+^ centre, though it must be in a strongly electron withdrawing environment. For instance, negative isomer shifts can be obtained in low-spin Fe^3+^ complexes in the presence of strong back-bonding (e.g., K_3_[Fe^3+^(CN)_6_] or Na_2_[Fe^3+^(CN)_5_NO])^74^. Additionally, when [Fe^3+^(CN)_6_]^3^^–^ is coordinated in supramolecular assemblies (e.g., Prussian blue analogues) involving metal–metal interactions. Such second-sphere coordination of the Fe–CN bonds by another metal ion can shift the Fe *δ* by ~–0.1 mm/s. Thus, metal–metal or charge transfer interactions in CoFeO_*x*_ could effectively decrease the Fe-based *s* electron density and give rise to a negative *δ* and high Fe K-edge energy. However, this scenario would result in an isomer shift that gradually shifts more negative as this Fe species is surrounded by more Co centres. This is not observed here; the isomer shift of the Fe species remains relatively constant at *δ* ~ –0.2 mm/s and in fact becomes slightly less negative with increasing Co concentration (Supplementary Fig. 7). Similarly, the Fe^4+^ XAS spectra obtained from linear combination fits using different Co:Fe ratios are very similar (Supplementary Fig. 9). These considerations, together with the weak ligand field imposed by oxide coordination and the consistency between the Mössbauer and Fe K-edge XAS data, suggest that the Fe species observed here can be assigned to a high-valent Fe^4+^ centre in an *O*_*h*_ ligand field.

## Discussion

Iron activates M-OECs for OER but its role appears to differ with the nature of the M-OEC and the condition under which it operates. Although most OER is performed in concentrated base, the large-scale deployment of renewable energy storage has prompted interest in performing OER in neutral water sources^75,76^. For this line of investigation, M-OECs excel owing to their stability arising from their self-healing properties^22^. At neutral pHs, Fe^3+^ plays a role in OER that appears to be derived from non-redox properties. PCET activation of water is impaired since water is a poor proton acceptor; Fe may act as a Lewis acid^77^ to increase the acidity of OH_*x*_ (aqua/hydroxo) moieties that are coordinated to M-OECs and thereby lower the reduction potential for the M^3+/4+^ couple and lead to a greater population of M^4+^ in the Fe-doped catalysts. This in turn gives rise to increased oxyl character (M(IV)⋯ O ↔ M(III)–O^•^). This Lewis acidity behaviour is supported by the observation that Fe doping in NiPbO_*x*_ shows no enhancement in OER at solution pH values commensurate with the p*K*_a_ of Fe^3+^. Moreover, OER enhancement may be replicated by non-redox active, Lewis acidic cations in Fe-free Ni-OECs^78^. In concentrated base, OH^–^ can adequately serve the role of a proton acceptor and the influence of the Fe^3+^ is diminished. When Fe^4+^ is implicated in OER, as has been proposed in numerous studies, OER appears to occur at the M (Co or Ni) metal centre with Fe^4+^ promoting the activation of OER at the M of the M-OEC. Such proposals are consistent with the electronic structure of first row transition metal centres confronting the “oxo-wall”^79^. Moving to the right in the periodic table, the *d*-electron count for the M^4+^ formal oxidation state increases and in a tetragonal oxide ligand field, the *d*_xz_ and *d*_yz_ orbitals are populated, preventing electron donation from terminal oxygen to the metal centre. Consequently, the M–O bond strength is much weaker for Co^4+^ than for Fe^4+^, which formally accommodates a kinetically more inert metal-oxo double bond. From a kinetics perspective, we believe that Fe^4+^ is not the active site from which OER occurs but rather OER occurs from Co centres with the Fe^4+^ participating as a redox cooperative centre where Fe^4+^ enhances the oxidizing power of a Co:Fe active site (Supplementary Fig. 1) versus a Co^4+^-only active site. Thus, we believe that for both NiFeO_*x*_ and CoFeO_*x*_ systems, Fe^3+^ functions as a Lewis acid in promoting PCET reactivity for the OER. However, unlike NiFeO_*x*_, OER activity in CoFeO_*x*_ tracks the Fe^4+^ alloying concentration, suggesting that the redox properties of the Co^4+^ centre is further enhanced by the presence of redox active Fe^4+^ centres.

In conclusion, we have spectroscopically detected and characterized a high-valent Fe^4+^ centre in CoFeO_*x*_ thin film OECs. Spectroscopic data suggest that this Fe^4+^ centre is located in a symmetric *O*_*h*_ oxide ligand field. The correlation between Fe^4+^ content and OER activity in CoFeO_*x*_ thin films suggests an important role of this high-valent state in the mechanism of O–O bond formation and oxygen evolution and supports the merits of exploring mixed-metallate oxygen evolution catalysts.

## Methods

### Materials

Catalysts with specific Fe:Co ratios were prepared by electrodeposition from metal nitrate salt solutions that were degassed. After deposition, the film was rinsed briefly in Type I water and then submerged in KOH buffer. Films were held at a constant potential of 0.84 V in 1 M KOH pH 14 or 1.0 V in 0.1 M KOH pH 13 for 3 h to convert the film to the oxyhydroxide form before further electrochemical analysis. To obtain films of various thicknesses, the total deposition time was altered between 30 and 120s and the current held during deposition was changed between 0.5, 1.0 and 5.0 mA/s. The exact film loading was obtained from ICP-MS analysis of the films.

### Electrochemistry

All electrochemical experiments were conducted at room temperature (23 ± 1 °C). Electrode potentials were converted to the NHE scale using *E*(NHE) = *E*(Ag/AgCl) + 0.197 V. Overpotentials for the OER from water were computed using η = *E*(NHE) − (1.23 V − 0.059 V × pH).

### Spectroscopy

CoFeO_x_ catalyst with natural ^57^Fe abundance were prepared for Mössbauer spectroscopy at 77 K. The data were calibrated and fit to linear combinations of symmetric pairs of Lorentzian peaks. Fe K-edge XANES spectra were collected at beamline 12BM-B at the Advanced Photon Source at Argonne National Laboratory. Reconstructed spectra of the pure Fe^3+^ and Fe^4+^ species were obtained through linear combinations of the XAS spectra of the various CoFeO_*x*_ films.

## Supplementary information

Supplementary Information

## Data Availability

Experimental procedures, characterization of compounds electrochemical and spectral data are available in the Supplementary Information. All data are available from the authors on reasonable request.
