# Supplementary material for: Detection of high-valent iron species in alloyed oxidic cobaltates for catalysing the oxygen evolution reaction
Source: Nat Commun. 2021 Jul 9;12:4218. doi: 10.1038/s41467-021-24453-6 (PMC8270959; doi:10.1038/s41467-021-24453-6)
Supplement: Supplementary file 1 — Supplementary Information [file 41467_2021_24453_MOESM1_ESM.pdf]

nature COMMUNICATIONS

Supporting Information

Detection of High-Valent Iron Species in Cobalt Oxides for Catalytic Oxygen Evolution

Nancy Li,[a] D. Ryan G. Hadt,*[b,c] Dugan Hayes,*[b,d] Lin X. Chen[b,e]
and Daniel G. Nocera[a,]*

[a] Department of Chemistry and Chemical Biology, Harvard University, Cambridge, MA 02138
[b] Chemical Sciences and Engineering Division, Argonne National Laboratory, Lemont, IL 60439
[c] Division of Chemistry and Chemical Engineering, California Institute of Technology, Pasadena, CA 91125
[d] Department of Chemistry, University of Rhode Island, Kingston, RI 02881
[e] Department of Chemistry, Northwestern University, Evanston, IL 60208

* Corresponding author email: dnocera@fas.harvard.edu, rghadt@caltech.edu, dugan@uri.edu

Table of Contents

A. Experimental Methods S3

A.1 Materials S3

A.2 Electrochemical methods S3

A.3 Electrolyte cleaning using chelating resin S3

A.4 Cathodic deposition of CoFeOOH film preparation S4

A.5 Potentiostatic Tafel data collection S4

A.6 Cyclic voltammetry S5

A.7 Inductively coupled plasma mass spectrometry for elemental analysis S5

A.8 Preparation of CoFeOOH powder samples S5

A.9 Zero-field ^{57}Fe Mössbauer spectroscopy S6

A.10 X-ray absorption spectroscopy data collection and analysis S6

B. Electrochemistry S7

Figure S1. Cyclic voltammetry CoFeOx films S7

Figure S2. Tafel slope of $CoFeO_x$ films S8

C. Mössbauer Spectroscopy S9

Figure S3. Zero-field ^{57}Fe Mössbauer spectra of CoFeOx films and fits (variable QS) S9

Figure S4. Zero-field ^{57}Fe Mössbauer spectra of CoFeOx films and fits (fixed QS) S10

Figure S5. Zero-field ^{57}Fe Mössbauer spectra CoFeOx films S11

Figure S6. Population of Fe^{3+} and Fe^{4+} species in $CoFeO_x$ films S12

Figure S7. Isomer shift and quadrupole splitting from fits in Mössbauer spectra S13

D. X-Ray Absorption Spectroscopy S14

Figure S8. Fe K-edge, k-space, and R-space of $CoFeO_x$ films S14

Figure S9. Fe K-edge, k-space, and R-space of calculated Fe^{3+} and Fe^{4+} S15

Figure S10. Fe K-edge, k-space, and R-space of 50%Fe:50%Co film S16

E. References S17

A. Experimental Methods

A.1 Materials

$Co(NO_3)_2 \cdot 6H_2O$ (99.999%) and $FeCl_2 \cdot 4H_2O$ (99%) were used as received from Strem. KOH (88%) was reagent grade and used as received from Macron. 65-71% TraceSELECT Ultra nitric acid, TraceSELECT cobalt and iron standard for ICP were used as received from Fluka Analytical. Chelex® 100 Resin was obtained from Bio-Rad. All electrolyte solutions were prepared with type I water (EMD Millipore, 18.2 MΩ cm resistivity). Air duster was purchased from VWR. Boron nitride (98%) was used as received from Sigma Aldrich. Unless stated otherwise, the working electrode was a fluorine-doped tin-oxide (FTO; TEC-7) coated glass purchased as pre-cut 1 cm × 2.5 cm glass pieces from Hartford Glass. Prior to use, FTO slides were cleaned by sonication in acetone and then rinsed with Type I water. A 1 cm^2 geometric electrode area was created by masking the FTO with Scotch tape. An Ag/AgCl reference electrode was positioned close to the FTO in the working compartment, and a Pt mesh electrode in the auxiliary side of the H-cell was used to complete the circuit.

A.2 Electrochemical Methods

All electrochemical experiments were conducted using a CH Instruments 760C or 760D bipotentiostat, a BASi Ag/AgCl or a CH Instrument CHI 152 Hg/HgO reference electrode, and a Pt-mesh counter electrode in a three-electrode electrochemical cell configuration with a porous glass frit separating the working and auxiliary compartments. Measurements were performed at room temperature (23 ± 1 °C). Electrode potentials were converted to the NHE scale using $E(NHE) = E(Ag/AgCl) + 0.197$ V. Overpotentials for the oxygen evolution reaction from water were computed using $\eta = E(NHE) - (1.23\ V - 0.059\ V \times pH)$.

A.3 Electrolyte Cleaning Using Chelating Resin

KOH electrolyte solutions were cleaned by treating the electrolyte with regenerated Chelex® 100 Resin (Bio-Rad, Catalog #210011676) according to published protocol (*1*) with slight modifications. Approximately 15 g of resin was used to clean ~400 mL of buffer at a time. Chelex resin was first washed in 3 M TraceSELECT HNO_3 and then rinsed with 4 L of type I water. All solutions were

stirred in 1 L polystyrene bottles and filtered through clean Corning polystyrene 0.45 µM filters. Chelex resin was then regenerated by stirring the resin for 12 h in 1 M TraceSELECT HCl followed by rinsing with 5 L of type I water. The resin was then stirred with 1 M KOH (99.99%, semiconductor grade, Sigma-Aldrich) for 24 h at 60 °C. Chelex resin was then rinsed with type I water until the pH of the filtrate was that of the electrolyte to be cleaned (pH 14 for 1 M KOH, and pH 13 for 0.1 M KOH). The prepared resin was then stirred with the electrolyte to be cleaned for at least 72 h before being filtered and kept in clean polystyrene bottles previously washed with 2% nitric acid. pH measurements of buffers before and after resin cleaning confirm negligible pH changes.

A.4 Cathodic Deposition of CoFeOOH Film Preparation

A solution of 100 mM total metal content was prepared with a desired ratio of Fe:Co. To minimize precipitation of $Co(OH)_2$ and $Fe(OH)_3$, all solutions were degassed with Ar gas for 20 min prior to addition of metal salts. A three-electrode electrochemical cell was set up in a Teflon beaker with an FTO working electrode, Ag/AgCl reference electrode, and a Pt counter electrode in a glass frit. Unless stated otherwise, a cathodic current of 0.1 mA/s was passed through for 30 s for film deposition. After deposition, the film was rinsed briefly in Type I water and then submerged in KOH buffer. Films were then held at a constant potential of 0.84 V in 1 M KOH pH 14 or 1.0 V in 0.1 M KOH pH 13 for 3 h to convert the film to the oxyhydroxide form before further electrochemical analysis. To obtain films of various thicknesses, the total deposition time was altered between 30-120s and the current held during deposition was changed between 0.5, 1.0 and 5.0 mA/s. The exact film loading was obtained from ICP-MS analysis of the films.

A.5 Potentiostatic Tafel Data Collection

Current-potential data were obtained by conducting controlled potential electrolysis in KOH electrolyte at a variety of applied potentials. Prior to film preparation, the solution resistance was measured in the electrolysis bath to be used for Tafel data collection using the iR test function. Following film preparation, the working electrode was rinsed in fresh Co-free electrolyte and transferred, without drying, to the same electrolysis bath from which the solution resistance was measured. The electrode was allowed to equilibrate with the electrolysis solution for 100 s while being held at the first potential. The solution was stirred and steady-state currents were then

measured at applied potentials for 100s that descended from 0.85 V to 0.65 V in 1 M KOH pH 14 proceeding in 10 mV steps. Higher potentials were avoided to prevent too much O_2 production to physically damage the film and resulting in mass transport limits. Lower potentials were also avoided to prevent reduction of Co centers to Co^{3+} or Co^{2+}. The solution resistance measured prior to the data collection was used to correct the Tafel plot for ohmic potential losses.

A.6 Cyclic Voltammetry

Catalyst films were prepared and anodized as described above. CV scans taken in 0.1 M KOH were held at open circuit potential (~0.9 V) for 10 s and then immediately scanned towards positive potentials until a current of ~5 mA was reach at which point the direction of scan was reversed towards –0.2 V and reversed again towards positive potentials terminating at 1.0 V. All CV scans were corrected for ohmic potential losses.

A.7 Inductively Coupled Plasma Mass Spectrometry for Elemental Analysis

Trace elemental analysis was carried out with quadrupole ICP-MS (Thermo Electron, X-Series ICP-MS with collision cell technology). All pipettes and polypropylene tubes were soaked in ~5% TraceSELECT nitric acid overnight and rinsed with type I water. All pipette tips were pre-rinsed with 2% double-distilled trace nitric acid prior to use. Films were digested with 4 mL of 2% double distilled trace nitric acid. Film samples along with standards and controls were then scanned twice for 60 s each for ^{59}Co, and ^{56}Fe. Internal standards containing 50 ppb for Ge, and 25 ppb for In, Rh, and Bi in 5% HNO_3 were added to each sample and control samples containing 150 ppb of Co and Fe dispersed throughout the samples to confirm no signal drift.

A.8 Preparation of CoFeOOH Powder Samples

All films were deposited and anodized in a portable chamber purged with Ar gas. A 2 L beaker was used to enclose a solution containing 100 mM total metal content, a 11 cm x 25 cm x 3 mm FTO, a carbon cloth counter electrode, and a Ag/AgCl reference electrode. Deposition by controlled potential electrolysis was carried out on solutions at –1.3 V for 1 h while stirring at 100 rpm. After deposition, the film was rinsed briefly in Type I water and then submerged in resin cleaned 0.1 M KOH buffer (1.8 L of KOH in 2 L beaker). Films were then held at a constant potential of 0.84 V with

iR compensation for 5 h with stirring to convert films to metal oxyhydroxides. After anodization, films were dried off using an air duster and scrapped off of the FTO substrate using a razor blade. Powder samples were stored in the dark to prevent photoreduction.

A.9 Zero-field ^{57}Fe Mössbauer Spectroscopy

Large quantities of CoFeOx catalyst (~20 mg) with natural ^{57}Fe abundance were prepared by scaling up the cathodic deposition and subsequent anodization. The resulting films were dried by pressurized air at room temperature and scraped off the electrode for analysis by zero-field ^{57}Fe Mössbauer spectroscopy at 77 K. Solid samples (~ 20 mg) were restrained with Paratone-N oil. The data was measured with a constant acceleration spectrometer (SEE Co., Minneapolis, MN). Isomer shifts are given relative to α-Fe metal at 298 K. The data was calibrated and fit to linear combinations of symmetric pairs of Lorentzian peaks. First, the fits were performed allowing the amplitude, isomer shift, quadrupole splitting, and linewidth of each of the two species to vary independently for each sample; these fits are shown in Figure S3. Then, to further restrain the fits and reduce the number of variable parameters, the fits were performed using fixed quadrupole splittings of 0.13 and 0.70 mm/s for the Fe^{4+} and Fe^{3+} species, respectively. These splittings were taken from the fully-variable fits of the 20%Fe:80%Co and 90%Fe:10%Co samples, as these contained the highest relative populations of Fe^{3+} and Fe^{4+}, respectively. These fits are shown in Figure S4. Both procedures yielded qualitatively similar results, and all fitting parameters are plotted as a function of Fe mol% in Figures S5 and S6.

A.10 X-ray Absorption Spectroscopy Data Collection and Analysis

Fe K-edge (7.112 keV) X-ray absorption near-edge structure (XANES) spectra were collected at beamline 12BM-B at the Advanced Photon Source at Argonne National Laboratory using a water-cooled, double-crystal, fixed-exit X-ray monochromator with Si(111) crystals and a double mirror system (flat plus toroidal) with a cutoff energy of 23 keV to focus the beam to ~0.5 mm (v) x 1 mm (h). All data were collected in transmission mode. In this setup, an ion chamber was placed before the sample for the incident X-ray flux reference signal I_0, and a second and third ion chamber were placed after the sample. An Fe foil was inserted between the second and third ion chambers for energy calibration. XAS data were collected at room temperature on $CoFeO_x$ powder samples

suspended in boron nitride contained by Kapton tape. No sample damage due to X-ray beam exposure was observed after multiple scans using the same sample position. Background subtraction and data normalization were carried out using the Athena software package (*2*).

Reconstructed spectra of the pure Fe^{3+} and Fe^{4+} species were obtained through linear combinations of the XAS spectra of the various $CoFeO_x$ films. The spectra of the 20%Fe:80%Co and 90%Fe:10%Co films were chosen as the most representative of the Fe^{4+} and Fe^{3+} species, respectively, and the spectra shown in Figure 3 were obtained subtracting a fraction of one spectrum from the other and rescaling, with the subtraction and scaling factors determined from the relative populations obtained from the fits of the zero-field ^{57}Fe Mössbauer spectra of films of the same composition. The same analysis was performed with the XAS spectra of other films (Figure S9), and the results show excellent qualitative agreement.

B. Electrochemistry

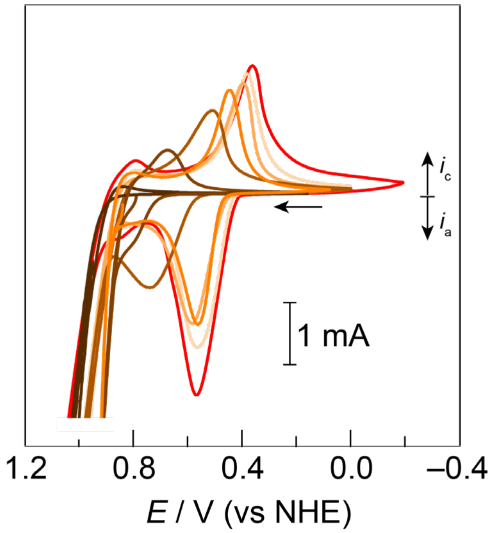

Figure S1. Cyclic voltammetry in resin cleaned 0.1 M KOH of $CoFeO_x$ films with Fe content: 0%(▬), 10% (▬), 20% (▬), 40% (▬), 60% (▬), 80% (▬), and 90% (▬). Scan rate: 0.1 V/s.

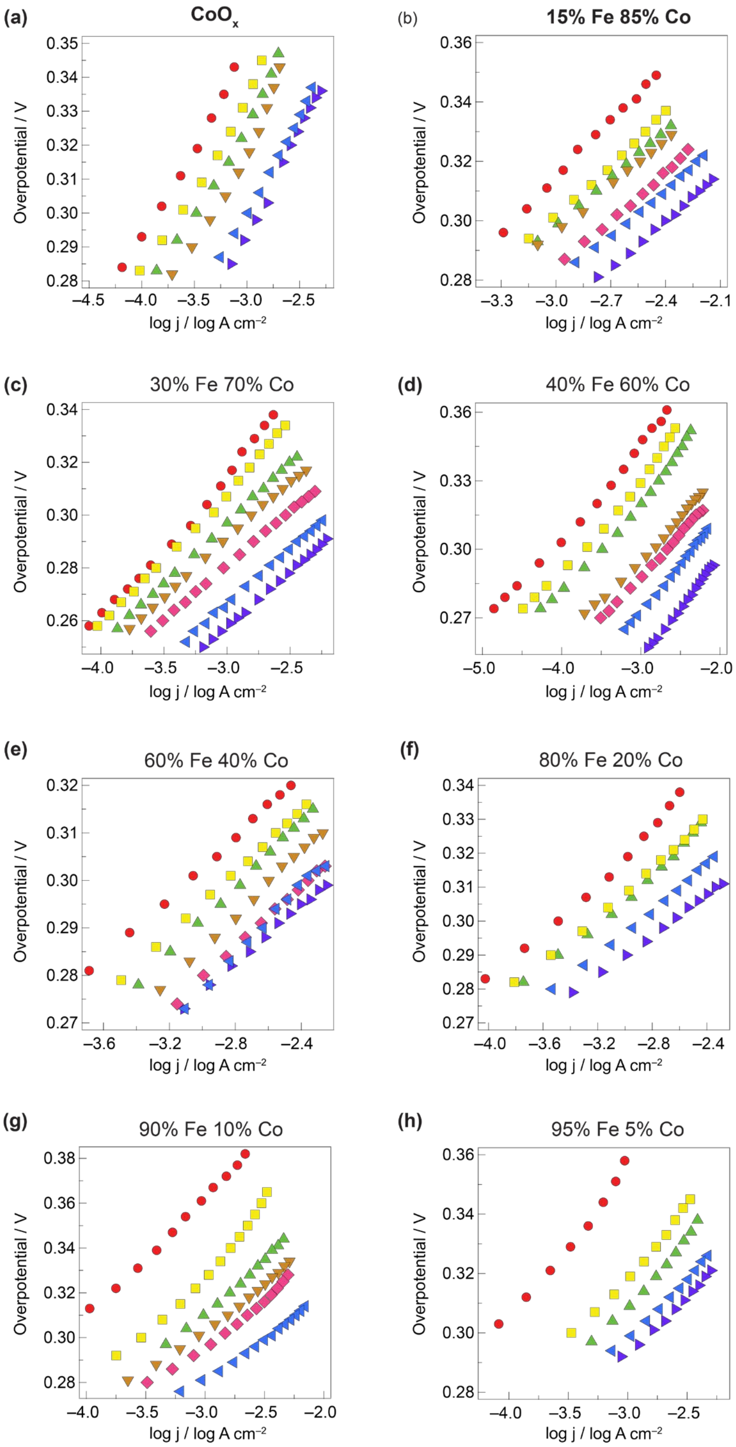

Figure S2. Tafel slope of $CoFeO_x$ films in resin cleaned 0.1 M KOH of increasing thickness with Co:Fe compositions: (a) 100% Co, (b) 15% Fe 85% Co, (c) 30% Fe 70% Co, (d) 40% Fe 60% Co, (e) 60% Fe 40% Co, (f) 80% Fe 20% Co, (g) 90% Fe 10% Co, and (g) 95% Fe 5% Co. The points on the graph vary from 15 nmol (red circle, ●) to 1000 nmol (purple triangle, ▶) total metal loading.

C. Mössbauer Spectroscopy

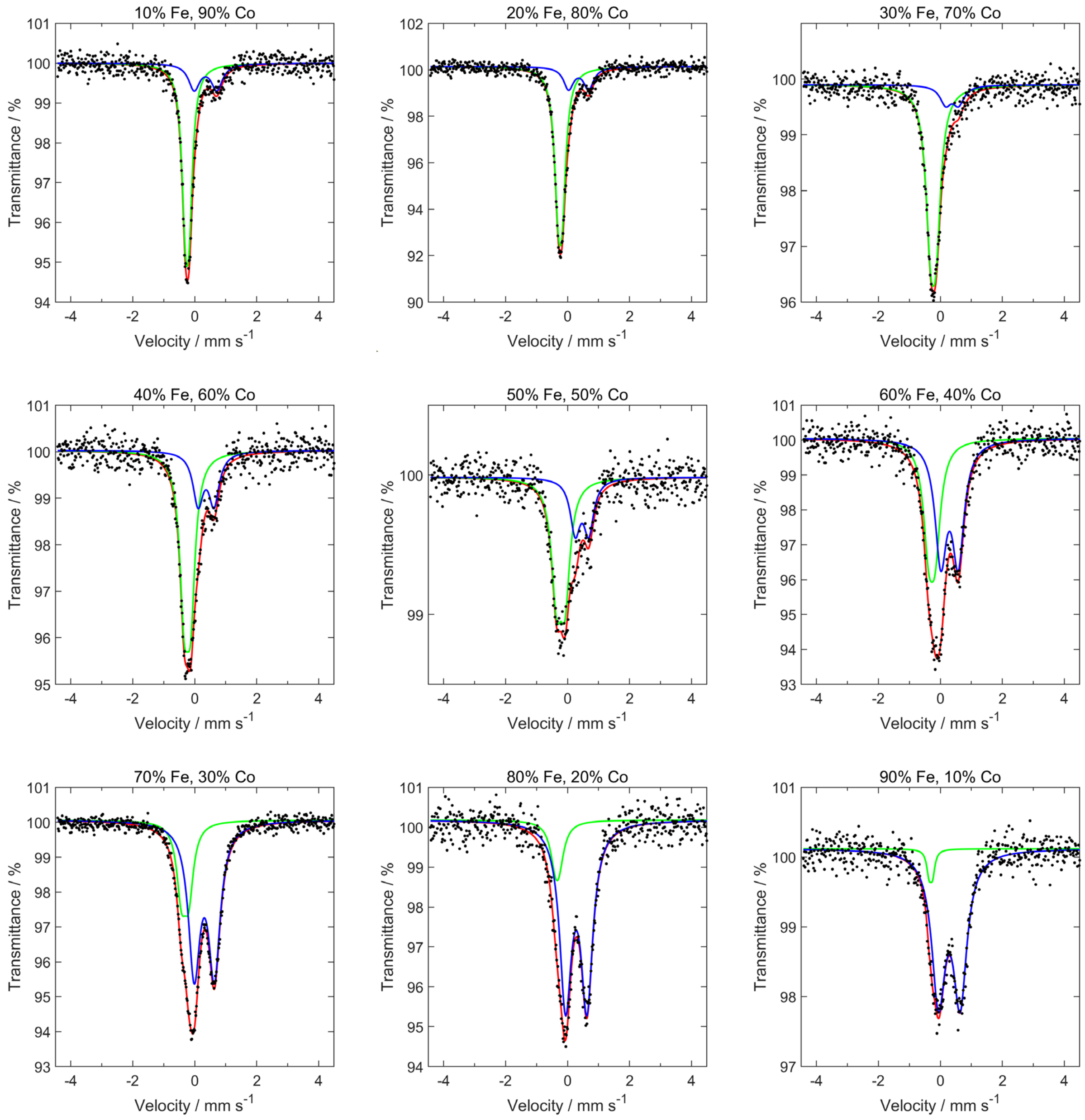

Figure S3. Zero-field ^{57}Fe Mössbauer spectra for $CoFeO_x$ films with increasing iron composition fit with the quadrupole splittings of the two iron species as variable parameters. Raw data (•), fit for Fe^{3+} species (blue), fit for Fe^{4+} species (green), and overall fit (red).

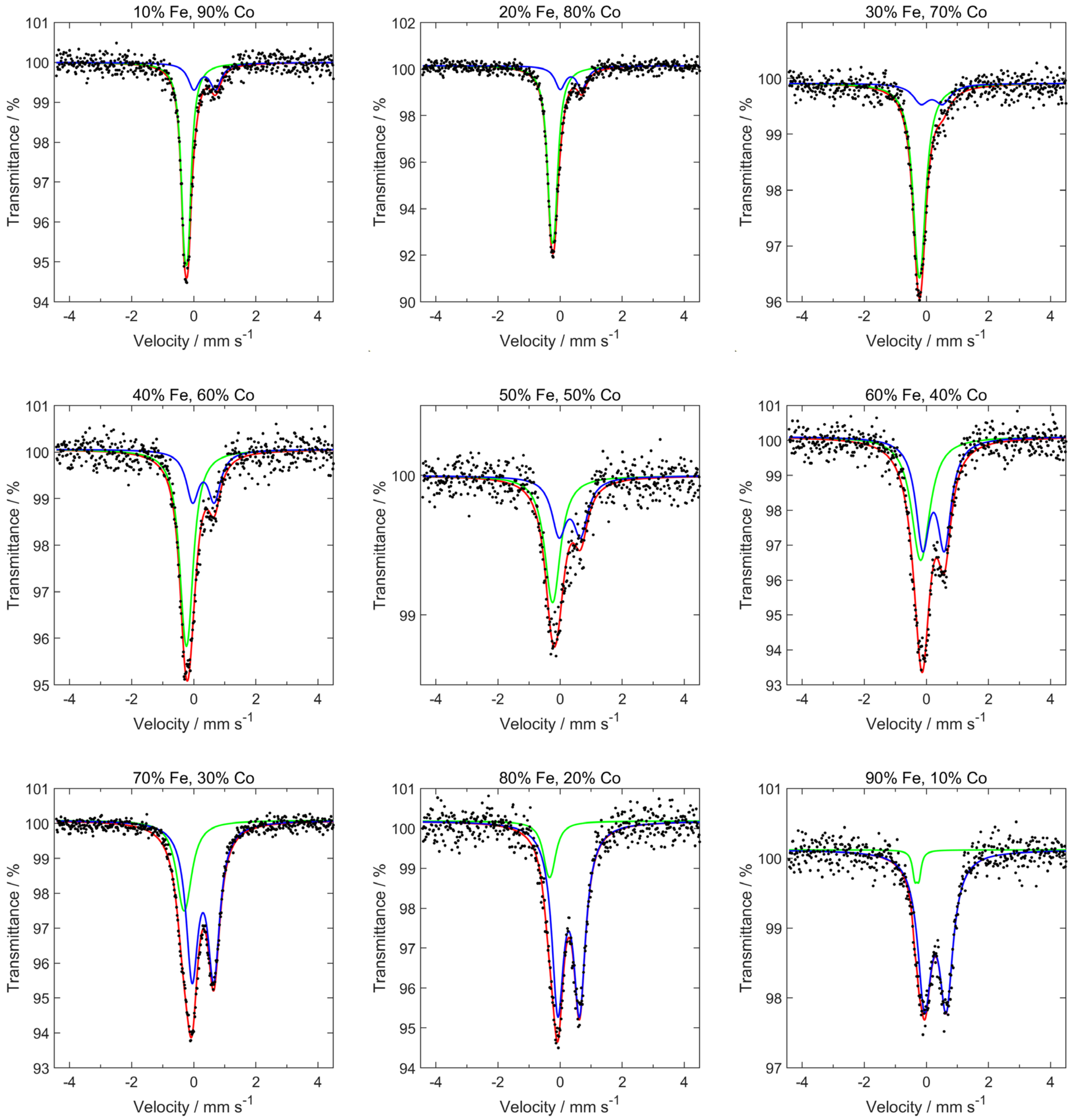

Figure S4. Zero-field ^{57}Fe Mössbauer spectra for $CoFeO_x$ films with increasing iron composition fit with the quadrupole splittings of the Fe^{3+} and Fe^{4+} species fixed at 0.13 and 0.70 mm/s, respectively. Raw data (•), fit for Fe^{3+} species (▬), fit for Fe^{4+} species (▬), and overall fit (▬).

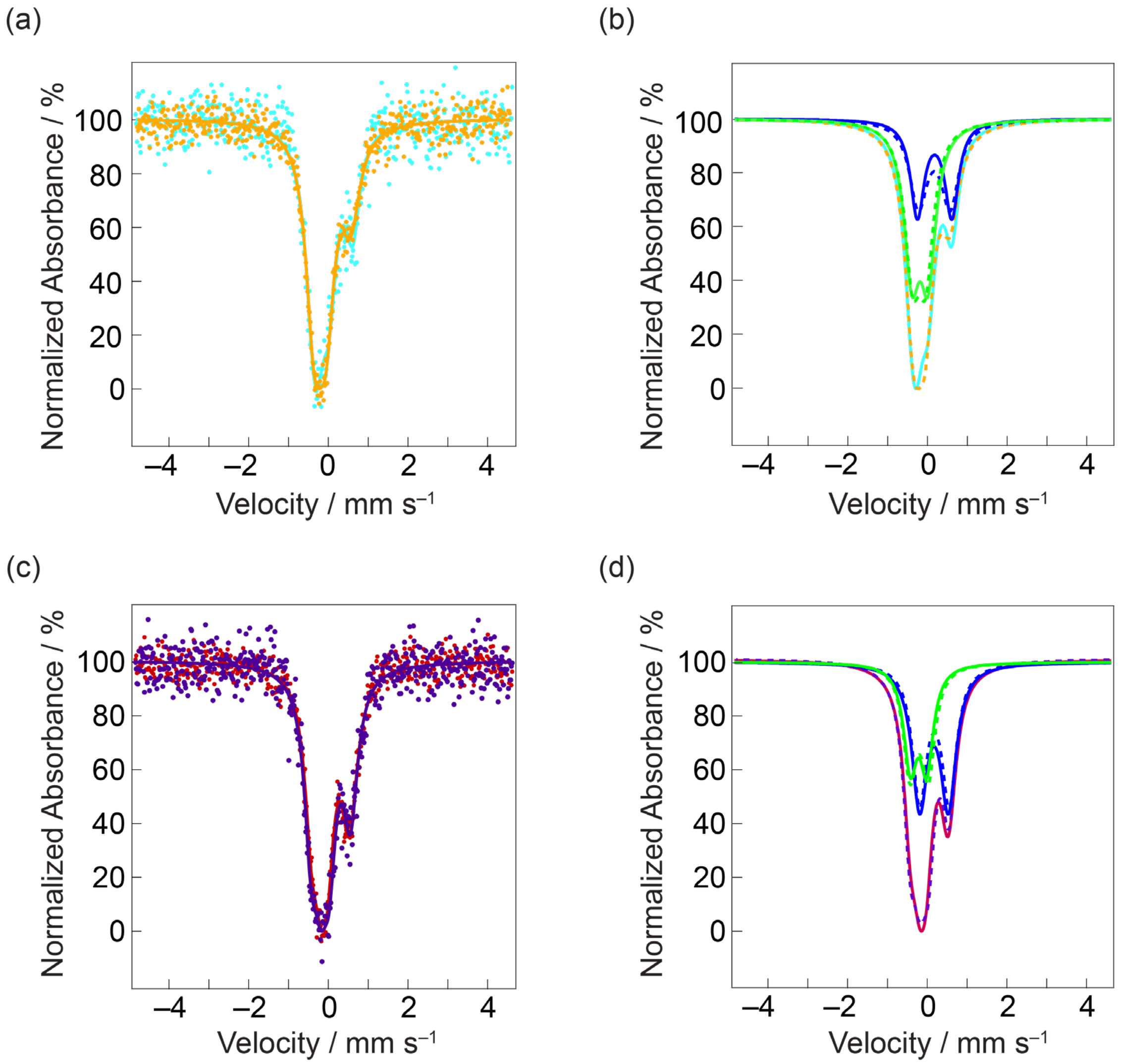

Figure S5. (a) Zero-field ^{57}Fe Mössbauer spectra for 50%Fe:50%Co $CoFeO_x$ of two independent samples (raw data: sample 1 (•) and sample 2 (•), overall fit: sample 1(—) and sample 2 (—)), and (b) corresponding fit to Fe^{3+} species (sample 1 (—) and sample 2(– –)), Fe^{4+} species (sample 1(—) and sample 2(– –)), and overall fit (sample 1(—) and sample 2(– –)). (c) Zero-field ^{57}Fe Mössbauer spectra for 60% Fe: 40% Co $CoFeO_x$ of two independent samples (raw data: sample 1 (•) and sample 2 (•), overall fit: sample 1(—) and sample 2 (—)), and (b) corresponding fit to Fe^{3+} species (sample 1 (—) and sample 2(– –)), Fe^{4+} species (sample 1(—) and sample 2(– –)), and overall fit (sample 1(—) and sample 2(– –)).

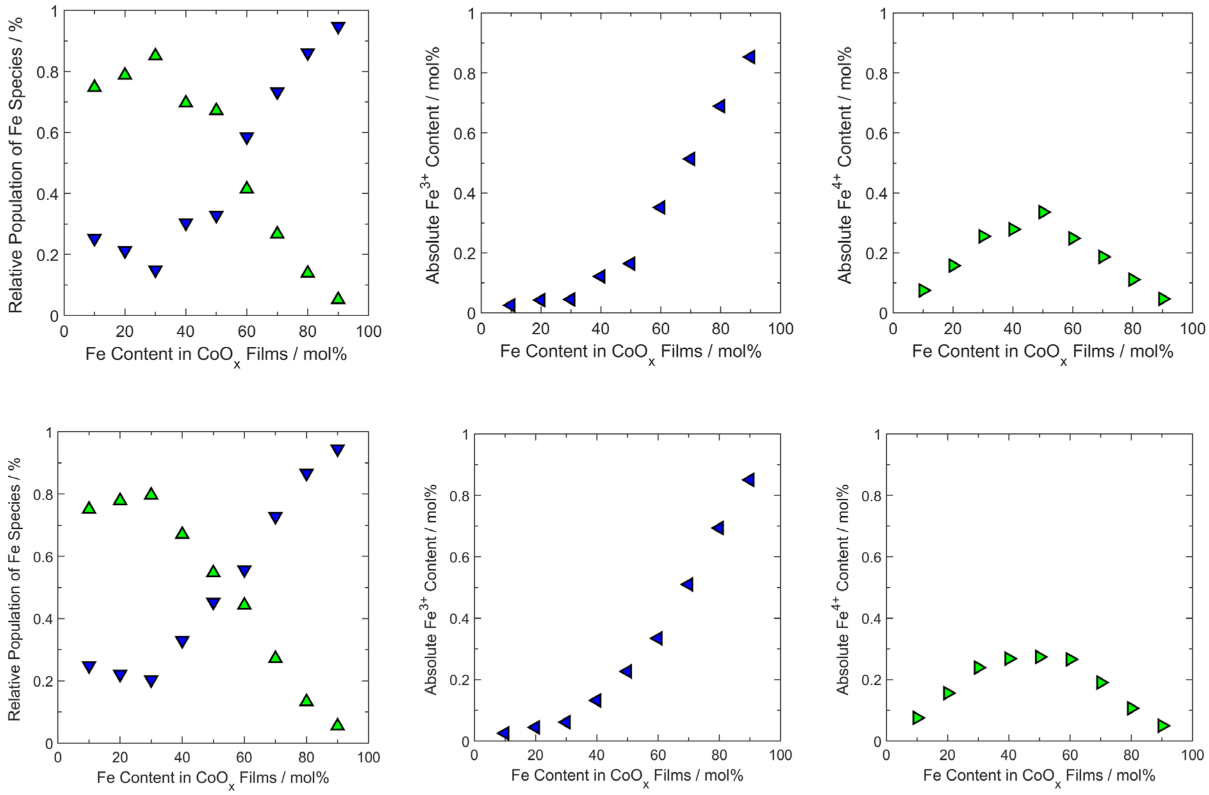

Figure S6. Relative population of Fe^{3+} (▼) and Fe^{4+} (▲) species (left) and absolute population of Fe^{3+} species (center, ◀) and Fe^{4+} species (right, ▶) extracted from fits of the zero-field ^{57}Fe Mössbauer spectra of $CoFeO_x$ films with increasing Fe content with the quadrupole splittings of the two iron species as variable parameters (top) and with the quadrupole splittings of the two iron species fixed at 0.13 and 0.70 mm/s, respectively (bottom). See text for description of calculations.

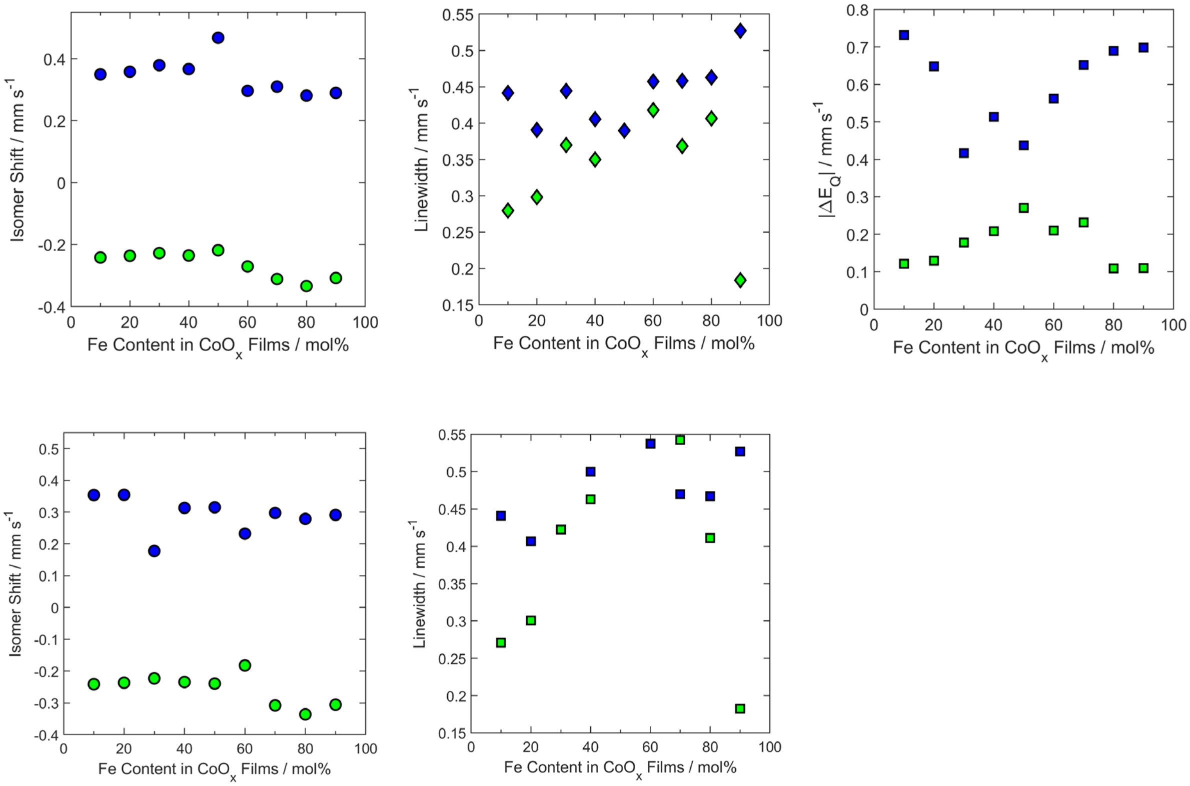

Figure S7. Isomer shifts (left, circles), linewidths (center, diamonds), and quadrupole splittings (right, squares) of the Fe^{3+} (blue) and Fe^{4+} (green) species extracted from fits of the zero-field ^{57}Fe Mössbauer spectra of $CoFeO_x$ films with increasing Fe content with the quadrupole splittings of the two iron species as variable parameters (top) and with the quadrupole splittings of the two iron species fixed at 0.13 and 0.70 mm/s, respectively (bottom).

D. X-Ray Absorption Spectroscopy

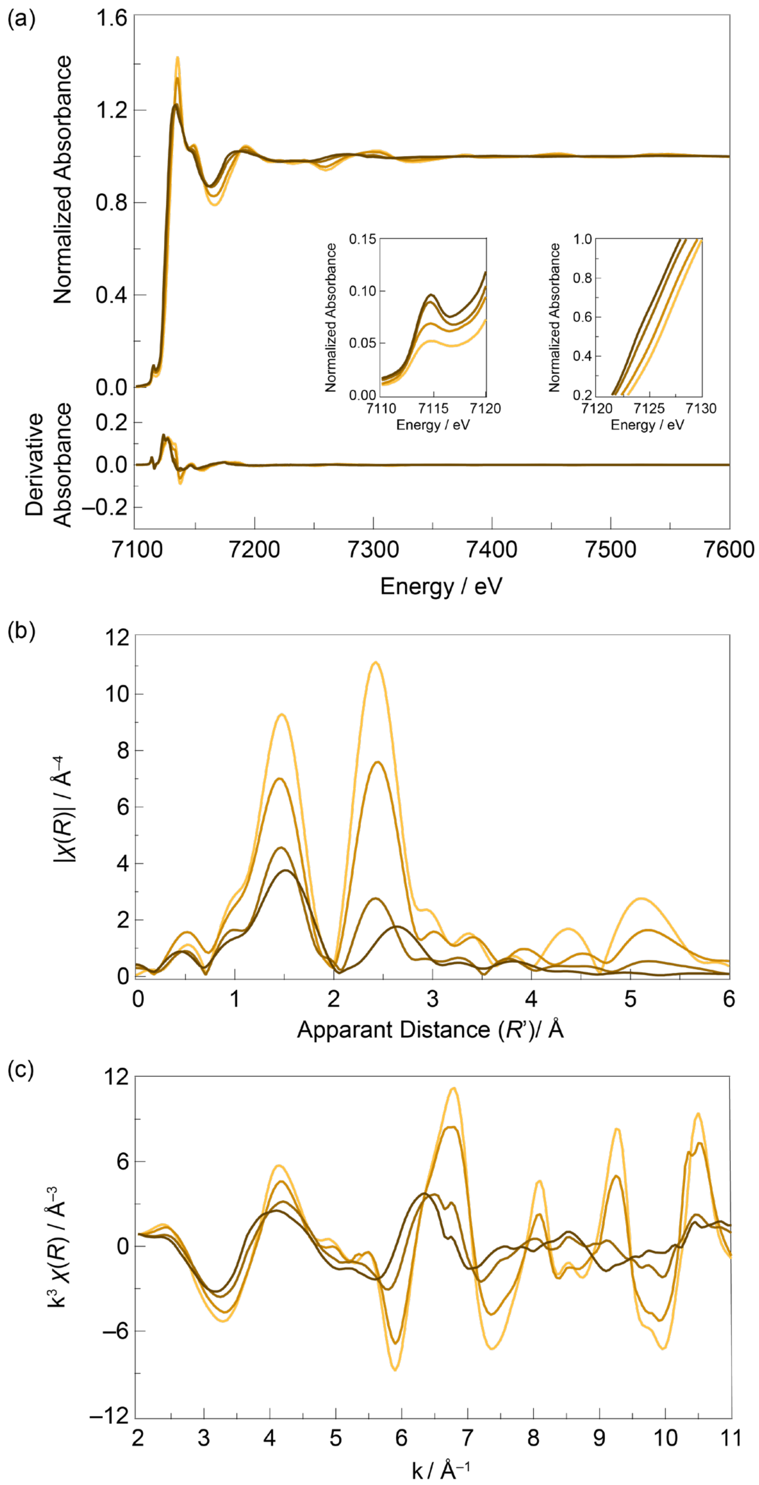

Figure S8. (a) Fe K-edge XANES spectra of $CoFeO_x$ films with the composition: 20%Fe:80% Co (▬), 50%Fe:50%Co (▬), 70%Fe:30%Co (▬), 90%Fe:10%Co (▬). Fourier transform of Fe K-edge EXAFS spectra (b) in R-space and (c) k-space.

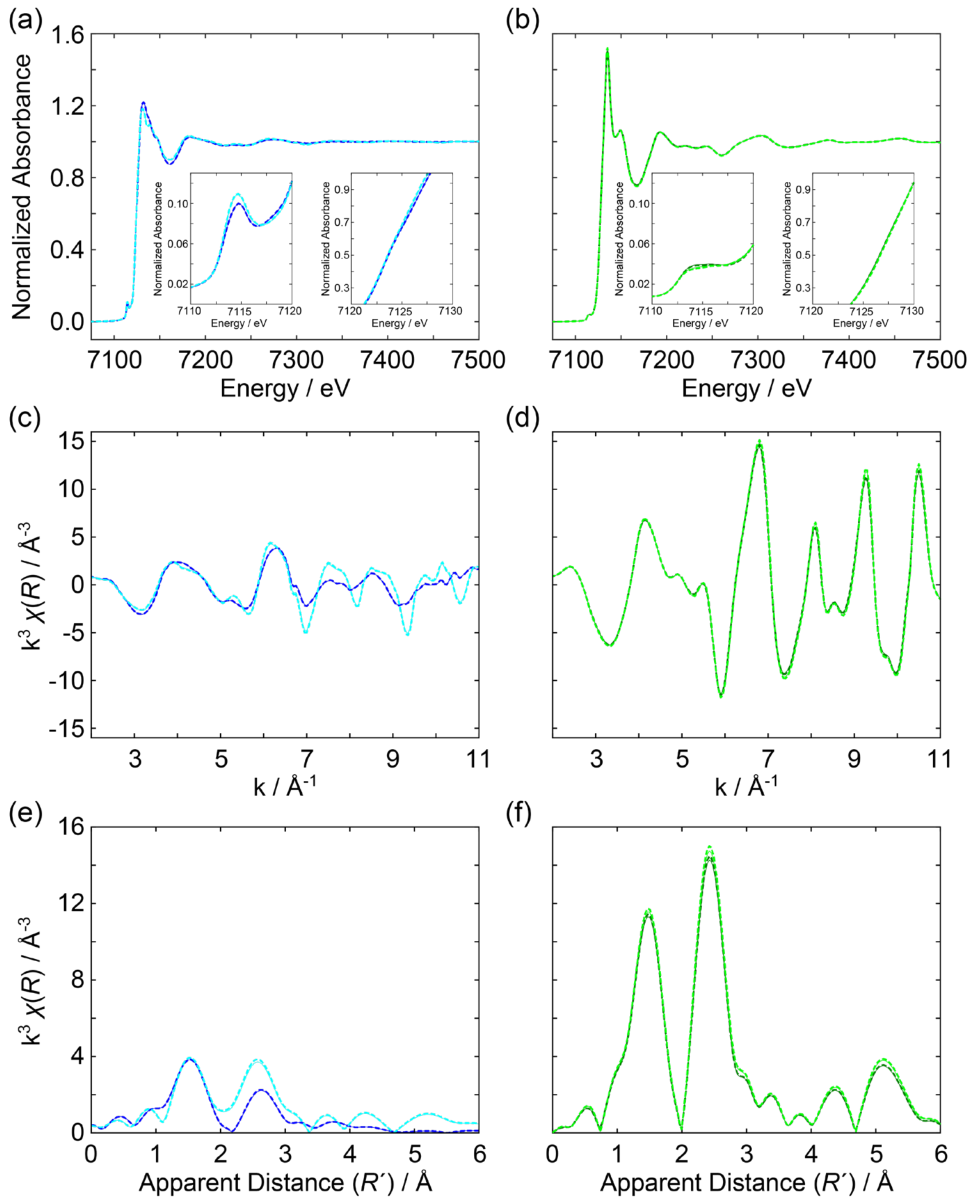

Figure S9. (a) Fe K-edge XANES spectra of Fe^{3+} calculated from: 20%Fe+80%Co/90%Fe+10%Co (▬), 20%Fe+80%Co/70%Fe+30%Co (▬), and Fe^{4+} calculated from: 70%Fe+30%Co/20%Fe+80%Co (▬), 70%Fe +30%Co/20%Fe+80%Co (▬). Solid lines were calculated using relative populations obtained from fits of the zero-field ^{57}Fe Mössbauer spectra with the quadrupole splittings of the two iron species as variable parameters, while dashed lines were calculated using relative populations obtained from fits with the quadrupole splittings of the two iron species fixed at 0.13 and 0.70 mm/s, respectively. Corresponding Fe K-edge EXAFS spectra in (b) k-space and (c) R-space. The relative populations obtained from the different Mössbauer fitting procedures have essentially no effect on the spectra, and the solid and dashed lines are nearly indistinguishable. Some differences are seen in the Fe^{3+} spectra obtained from analysis of the spectra of different films, but the relative pre-edge intensity, edge energy, and first-shell R-space peak are essentially unchanged.

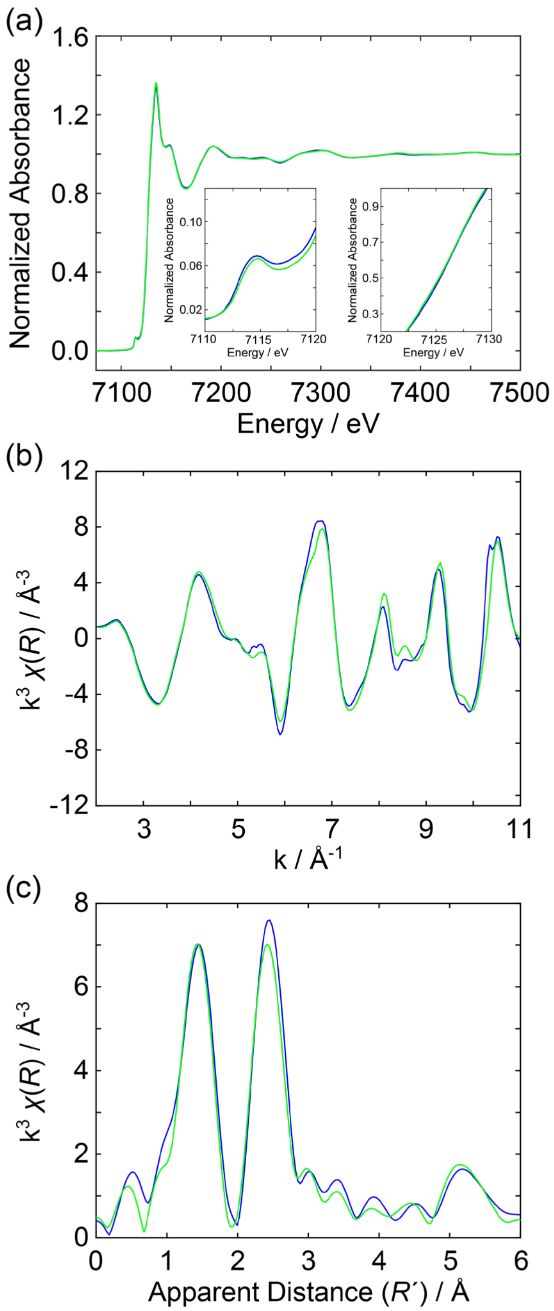

Figure S10. (a) Fe K-edge XANES spectrum of 50%Fe+50%Co film (▬) and linear combination spectrum calculated from the extracted Fe^{3+} and Fe^{4+} spectra shown in Figure S9 (▬). The linear combination coefficients were 0.45 and 0.55 for Fe^{3+} and Fe^{4+}, respectively, as determined from the fit of the zero-field ^{57}Fe Mössbauer spectrum of the 50%Fe+50%Co film with the quadrupole splittings of the two iron species fixed at 0.13 and 0.70 mm/s, respectively (see Figures S4 and S6). The Fe^{3+} and Fe^{4+} spectra obtained from analysis of the 20%Fe+80%Co/90%Fe+10%Co XAS spectra were used for the linear combination. Corresponding Fe K-edge EXAFS spectra in (b) k-space and (c) R-space. All experimental and calculated spectra show excellent agreement, providing further validation of our combined ^{57}Fe Mössbauer and Fe K-edge XAS analysis.

E. References

1. A. Wuttig & Y. Surendranath, Impurity ion complexation enhances carbon dioxide reduction catalysis. *ACS Catal.* **5**, 4479–4484 (2015).
2. B. Ravel & M. Newville, ATHENA, ARTEMIS, HEPHAESTUS: data analysis for X-ray absorption spectroscopy using IFEFFIT. *J. Synchrotron. Radiat.* **12**, 537–541 (2005).
